# Sex-specific differences in atherosclerosis, thrombospondin-1, and smooth muscle cell differentiation in metabolic syndrome versus non-metabolic syndrome mice

**DOI:** 10.3389/fcvm.2022.1020006

**Published:** 2022-11-23

**Authors:** Shreya Gupta, Saugat Khanal, Neha Bhavnani, Amy Mathias, Jason Lallo, Ariana Kiriakou, Jessica Ferrell, Priya Raman

**Affiliations:** ^1^Department of Integrative Medical Sciences, Northeast Ohio Medical University, Rootstown, OH, United States; ^2^School of Biomedical Sciences, Kent State University, Kent, OH, United States; ^3^Department of Biological Sciences, Kent State University, Kent, OH, United States

**Keywords:** TSP-1, metabolic syndrome, SMC differentiation, atherosclerosis, KKAy mice, sex-specific differences

## Abstract

**Introduction:**

Metabolic syndrome (MetS) amplifies the risks of atherosclerosis. Despite well-known sexual dimorphism in atherosclerosis, underlying mechanisms are poorly understood. Our previous findings highlight a proatherogenic protein, thrombospondin-1 (TSP-1), in hyperglycemia- or hyperleptinemia (mimicking obesity)-induced atherosclerosis. However, the role of TSP-1 in the development of atherosclerosis prompted by co-existing hyperglycemia and obesity, characteristic of MetS, is unknown. The goal of this study was to examine sex-specific differences in lesion progression in a model of combined MetS and atherosclerosis (KKAyApoE) and interrogate how these differences relate to TSP-1 expression.

**Methods:**

Male and female KKAy^+/−^ApoE^–/–^ (with ectopic agouti gene expression) and age-matched non-agouti KKAy^–/–^ApoE^–/–^ littermates were placed on a standard laboratory diet from 4 to 24 weeks age followed by blood and tissue harvests for biochemical, molecular, and aortic root morphometric studies.

**Results:**

Metabolic profiling confirmed MetS phenotype of KKAy^+/−^ApoE^–/–^; however, only male genotypes were glucose intolerant with elevated VLDL-cholesterol and VLDL-triglyceride levels. Aortic root morphometry demonstrated profound lipid-filled lesions, increased plaque area, and augmented inflammatory and SMC abundance in MetS vs non-MetS males. This increase in lesion burden was accompanied with elevated TSP-1 and attenuated LMOD-1 (SM contractile marker) and SRF (transcriptional activator of SM differentiation) expression in male MetS aortic vessels. In contrast, while lipid burden, plaque area, and TSP-1 expression increased in MetS and non-MetS female mice, there was no significant difference between these genotypes. Increased collagen content was noted in MetS and non-MetS genotypes, specific to female mice. Measurement of plasma testosterone revealed a link between the atherogenic phenotype and abnormally high or low testosterone levels. To interrogate whether TSP-1 plays a direct role in SMC de-differentiation in MetS, we generated KKAy^+/−^ mice with and without global TSP-1 deletion. Immunoblotting showed increased SM contractile markers in male KKAy^+/−^TSP-1^–/–^ aortic vessels vs male KKAy^+/−^TSP-1^+/ +^. In contrast, TSP-1 deletion had no effect on SM contractile marker expression in female genotypes.

**Conclusion:**

Together, the current study implicates a role of plasma testosterone in sex-specific differences in atherosclerosis and TSP-1 expression in MetS vs non-MetS mice. Our data suggest a sex-dependent differential role of TSP-1 on SMC de-differentiation in MetS. Collectively, these findings underscore a fundamental link between TSP-1 and VSMC phenotypic transformation in MetS.

## Introduction

Cardiovascular disease is the leading cause of morbidity and mortality world-wide accounting for nearly 19 million deaths in 2020, and this number is expected to escalate by 2030 (1). Atherosclerosis is a major player in the development of several cardiovascular complications including myocardial infarction, heart failure and stroke (1). Current lipid-lowering therapies, including the gold-standard statins, have provided limited benefit against major macrovascular events and cardiovascular mortality (2); moreover, many of these agents have reported significant toxicity and side-effects associated with their use (3, 4). Metabolic disorders such as hyperglycemia, obesity and dyslipidemia have a devastating impact on vascular function. Numerous clinical studies and trials including animal data highlight hyperglycemia, a hallmark of diabetes, and obesity as independent risk-factors for atherosclerosis (5–13). Risk of atherosclerotic complications is amplified two-to-four-fold (14) in individuals with metabolic syndrome (MetS), a cluster of metabolic anomalies characterized by hyperglycemia and obesity. Despite extensive work, mechanisms responsible for accelerated atherosclerotic complications in MetS remain incompletely understood.

Earlier work from our laboratory demonstrates that high glucose, mimicking diabetes, and high leptin, mimicking obesity, upregulate the expression of a potent proatherogenic protein, thrombospondin-1 (TSP-1), in human and mouse aortic smooth muscle cells (SMC) (15, 16). TSP-1 is a multifunctional extracellular matrix protein that has been linked to metabolic disease and related cardiovascular complications (17). Growing literature indicates that TSP-1 expression is significantly increased in the plasma, visceral adipose tissue, heart, blood vessels and kidneys of patients with diabetes and obesity and related murine models (18–22). Earlier studies have also shown that TSP-1 expression is significantly enhanced in the injured vascular wall (23, 24) and atherosclerotic lesions (25). Additionally, multiple cell types within the vascular wall including endothelial cells, SMC, fibroblasts and macrophages produce TSP-1 in response to numerous proatherogenic stimuli (15, 16, 20). Recent studies from our laboratory have documented that global TSP-1 deletion *in vivo* reduces atherosclerotic lesion burden in ApoE^–/–^ mice in response to both STZ-induced hyperglycemia (26) and exogenous leptin administration (27), mimicking obesity. However, the role of TSP-1 in the development of atherosclerotic lesions under pathological conditions prompted by co-existing hyperglycemia and obesity, characteristic of MetS, remains elusive.

The pathophysiology of human atherosclerosis in MetS is highly complex, triggered by a combination of metabolic risk factors. Therefore, an understanding of the underlying molecular mechanisms that drive the disease requires animal models that most closely mimic the human state. While there are several genetically-modified murine models of diabetes and obesity such as db/db, and ob/ob mice, most of these existing mouse models do not fully represent the polygenic form of the disease and in turn, fail to recapitulate the complex spectrum of the human disease. Moreover, a plethora of growing literature emphasizes sex-specific differences in the incidence, clinical manifestations and etiology of atherosclerosis, including differential response to major cardiovascular risk factors (28–30). Despite the widely-accepted sexual dimorphism in atherosclerosis, mechanisms underlying sex as a biological variable in atherosclerosis are poorly understood. Such limitations have been largely attributed to the lack of well-powered, preclinical animal studies that encompass both male and female progenies.

Therefore, the objective of the current study was to examine sex-specific differences in the development of atherosclerosis between a murine model of MetS versus non-MetS developed on an atherosclerotic background, and to interrogate how these differences relate to TSP-1 expression in the vasculature. Our data suggest a putative link between plasma testosterone levels, atherogenic phenotype and TSP-1 expression in MetS vs non-MetS mice. These findings implicate a sex-specific differential role of TSP-1 on atherosclerotic lesion progression and SMC de-differentiation in MetS.

## Materials and methods

### Mouse models

All animal experiments and euthanasia procedures utilized in this study were conducted in accordance with animal protocols annually approved by Northeast Ohio Medical University Institutional Animal Care and Use Committee (NEOMED IACUC). ApoE^–/–^ (stock no. 002052) and TSP-1^–/–^ (stock no. 006141) mice on C57BL/6J background, and agouti KKAy^+/–^ (stock no. 002468) mice were purchased from The Jackson Laboratory (Bar Harbor, ME, USA) and further expanded in our animal facility. Generation of KKAy^+/–^ mice are known to involve transfer of the yellow agouti gene (Ay^+^) into the KK spontaneously diabetic mice by repeated crossing of the yellow obese mice with KK mice (black fur at weaning). Unlike the KK mice that carry only the diabetes gene, the KKAy^+/–^ mice have an ectopic expression of the agouti gene which blocks the melanocortin type 4 receptor signaling in the hypothalamus resulting in hyperphagic mice with a stronger expression of obesity than in the original KK mice (31). Male KKAy^+/–^ mice were crossbred with either female ApoE^–/–^ or female TSP-1^–/–^ mice. The resulting agouti KKAy^+/–^ApoE^–/–^ and non-agouti KKAy^–/–^ApoE^–/–^ as well as KKAy^+/–^/TSP-1^–/–^ and KKAy^+/–^TSP-1^+/+^ mice produced in F2 generation were utilized in this study. ApoE^–/–^ and TSP-1^–/–^ genotypes were confirmed by polymerase chain reaction based on established protocols, per The Jackson Laboratories. KKAy^+/–^ and KKAy^–/–^ genotypes were identified based on coat color, with yellow indicative of agouti Ay^+/–^ and black depicting non-agouti Ay^–/–^ allele transmission. Mice were housed in a pathogen-free environment and kept on a 12-hour:12-hour light/dark cycle.

### Study design

Male and female KKAy^+/–^ApoE^–/–^ and age-matched KKAy^–/–^ApoE^–/–^ littermate mice were weaned at 4 weeks of age and maintained on standard laboratory diet (Purina LabDiet 5008) *ad libitum* until 24 weeks of age (study end point). Body weight and non-fasted blood glucose levels were monitored in all animals on a monthly-basis. For agouti KKAy^+/–^ mice, animals with body weight <40 g and non-fasted blood glucose <200 mg/dl at 8-weeks-age were excluded from the study. Similarly, non-agouti KKAy^–/–^ genotypes with body weight >40 g and non-fasted blood glucose >200 mg/dl at 8 weeks of age were also excluded from the study. At 22 weeks of age, subsets of mice were subjected to either intraperitoneal glucose tolerance test (IPGTT) or insulin tolerance test (ITT) followed by Treadmill Exercise Test conducted at 23 weeks of age. After overnight fasting, animals were harvested at 24 weeks of age following euthanasia with Fatal Plus, per institutionally approved methods. Blood and tissue samples were collected and utilized for experiments as described below. In a parallel study, male and female KKAy^+/–^ TSP-1^+/+^ and age-matched KKAy^+/–^TSP-1^–/–^ littermates, weaned at 4-weeks-age, were maintained on standard laboratory diet (Purina LabDiet 5008) *ad libitum* until 18 weeks of age (study end point). Body weight and non-fasted blood glucose levels were monitored in all animals on a monthly basis. At 17 weeks of age, KKAy^+/–^TSP-1^+/+^ and KKAy^+/–^TSP-1^–/–^ mice were subjected to IPGTT; after overnight fasting, animals were harvested at 18 weeks of age for blood and tissue collection following euthanasia using Fatal Plus.

### Intraperitoneal glucose tolerance test and insulin tolerance test

After overnight fasting, an initial blood sample was collected from each mouse via lateral tail incision. This was followed by intraperitoneal injection of either sterile glucose solution (2 g/kg body weight) or sterile insulin (0.75 U/kg body weight) for glucose tolerance test (GTT) or insulin tolerance test (ITT), respectively. Thereafter, blood samples were collected at 15-, 30-, 60-, 90-, and 120-min post-glucose or insulin injections. All blood glucose measurements were made using a hand-held glucometer.

### Plasma lipid analyses

After an overnight fast, blood was collected from each animal via cardiac puncture; plasma was separated, aliquoted and stored at −80°C for future analyses. Total cholesterol and total triglyceride levels were measured in the plasma samples collected from each mouse using Infinity Reagents (Thermo Fisher Scientific, Waltham, MA, USA). Distribution of cholesterol and triglyceride among plasma lipoproteins was measured using fast-performance liquid chromatography (FPLC), as described earlier (27).

### Plasma testosterone assay

Total testosterone levels were measured in plasma samples collected from each mouse using the testosterone parameter assay kit (R&D Systems, Minneapolis, MN, USA), per manufacturer’s instructions.

### Treadmill exercise test

This test involves measurement of maximal oxygen consumption (VO_2max_) during exercise and was performed as reported earlier (32). One day prior to the experiment, mice were trained and acclimated to using the treadmill at walking speeds for 10–15 min. The next day, mice were allowed to run to exhaustion at incrementally increasing speeds ranging from 10 to 48 m/min at a constant incline of 5°. Briefly, mice were kept in sealed clear Plexiglass cages fitted with an automated conveyer belt used as a treadmill and shocker grid to stimulate running. Fresh room air was provided at 0.5 L/min for the duration of the acclimation and recording periods. On the day of the experimentation, initial baseline readings of oxygen consumption and carbon dioxide production were followed by measurement of gas exchange, maximum speed and maximum runtime until the animals reached exhaustion. Once the mice were exhausted and exposed to the shocker, they were removed from the apparatus and returned to their home cages. Care was taken to ensure that each mouse was not exposed to the shocker more than five times within a 1-min span; failure of the mouse to continue running determined termination of measurements for that mouse. Maximum oxygen consumption (VO_2max_), carbon dioxide production at the time of VO_2max_ (VCO_2max_), maximum running speed at which VO_2max_ was achieved, maximum run time until exhaustion and respiratory exchange ratio (RER [V_*CO*2_/V_*O*2_], an estimate of fuel usage) were calculated based on the readings and analyzed using Graph Pad.

### Aortic root morphometry

At the study endpoint, hearts were removed from each mouse following PBS perfusion. After a brief PBS wash, individual mouse hearts were embedded in OCT and stored at −80°C until further use. About 8–10-micron serial sections of the aortic root were obtained by cutting the OCT-embedded hearts, as described earlier (27). Care was taken to ensure that only serial sections collected from aortic root regions representing about 100–150 microns following the valve leaflet were utilized in all morphometric experiments. Additional care was exercised to ensure that staining was performed on sections within similar regions of the aortic root across all treatment groups for quantification and comparison. Aortic root sections were concurrently stained with 0.5% w/v Oil red O (ORO) and hematoxylin and eosin (H&E) to assess the lipid burden and plaque area, respectively. Additional sections were utilized for the detection of collagen content within aortic root lesions using Masson’s Trichrome stain kit (Polysciences). For ORO-stained and MT-stained sections, hematoxylin counterstaining was utilized. All sections were mounted with DPX mounting media, observed using Olympus BX61VS microscope and images were captured using 10× magnification. For quantitative morphometry, eight animals per genotype for each sex with at least 30 sections within each group were analyzed.

### Immunohistochemistry

Serial root sections acquired from each animal as described above were subjected to immunohistochemistry using CD68, CD45, and α-SMA (Abcam) antibodies, as described earlier (27). Briefly, tissue sections were incubated in ice-cold acetone (5–10 min) and blocked with 5% donkey or goat serum (60–90 min) at room temperature. Following an overnight incubation with primary antibodies (anti-CD68-1:800; anti-CD45-1:200; anti-α-SMA-1: 400) at 4°C, sections were incubated with donkey Alexa Flour 594 anti-rabbit IgG secondary antibodies (1:200) and mounted on DAPI-containing mounting media (Vectashield, Vector Laboratories, Newark, CA, USA). To control for non-specific staining, identical tissue sections were incubated with species-specific IgG control antibody in the absence of the corresponding primary antibodies or no primary antibody, wherein no background staining was noted. Sections were observed using Olympus fluorescence IX71 microscope (10× magnification) and images were digitally captured using a set of identical parameters across all sections, specific for each antibody. For all image quantifications, at least five mice per genotype for each sex with an average of 20 tissue sections within each group were utilized.

### Immunoblotting

Lysates from aortae of harvested animals were prepared in RIPA lysis buffer. Protein content was measured using the BCA protein assay (Thermo Fisher Scientific, Waltham, MA, USA). Equal concentrations of protein (25 μg of aortic lysates) were resolved on 8% SDS-PAGE followed by wet transfer to PVDF membranes. Immunoblotting was performed using anti-TSP-1 (1:200, Invitrogen, Waltham, MA, USA), anti-LMOD-1 (1:1000, ProteinTech, Rosemont, IL, USA), anti-SRF (1:1000, Cell Signaling, Danvers, MA, USA) and anti-Calponin (1.5:1000, Cell Signaling, Danvers, MA, USA). Equal protein loading of samples was confirmed by staining the membranes with Ponceau S. All immunoblot images were captured using Protein Simple and densitometric analyses was performed using Image J software. For representative immunoblots, lane images depict proteins loaded and detected on a single immunoblot; however, lanes were re-arranged to improve the clarity of presentation.

### Image quantification

For morphometry and immunohistochemistry studies, lesion area defined by the internal elastic lamina to luminal edge of the lesion was selected using the polygon selection tool in Image J; this region was cropped, saved as a new image file and subsequently used for analyses using Image J software. Briefly, the color thresholding option was utilized to measure the total lesion area and stained area for each image. For immunohistochemistry, specific positive staining was expressed as a percentage of the total lesion area. For measurement of plaque area in H&E-stained images, within each aortic root image, line tracings were drawn to mark the area enclosed by the internal elastic lamina (total aortic root area) and the area enclosed by the luminal edge of the lesions (aortic root luminal area). Plaque area was then determined by subtracting the aortic root luminal area from the total aortic root area for each H&E-stained image. All image quantifications were performed by team members blinded to the identity of all sections. For ORO and MT images, area was measured in μm^2^; for H&E images, area was measured in mm^2^; for CD45, CD68, and α-SMA images, area was measured in sq. pixels.

### Statistical analyses

All results are expressed as mean ± SEM. Statistical analyses was performed using GraphPad Prism 9.3.1 and Excel’s Real Statistics Resource pack. Prior to statistical analyses, each data set was tested for normality (Shapiro–Wilks; D’Agostino–Pearson; Kolmogorov–Smirnov; Cramer–von Mises; Ryan–Joiner) and variance on the original scale or after square root data transformation. Parametric methods were applied if assumption of normality was met, while non-parametric methods were used if the assumption was violated. For data following normal gaussian distribution with similar variance, ordinary one-way ANOVA followed by Tukey’s multiple correction *post-hoc* test was used for 4-group comparison. For normally distributed data with unequal variance, Welch correction was applied followed by Dunnett’s *post-hoc* test. Unpaired Student’s *t*-test was used for 2-group comparisons of normally-distributed data. Mann–Whitney and Kruskal–Wallis were applied as the non-parametric counterparts of Student’s *t*-test and one-way ANOVA, respectively; further, Kruskal–Wallis was followed by Dunn *post-hoc* test. Statistical significance was set at *p* ≤ 0.05.

## Results

### Metabolic profile of agouti KKAy^+/–^ApoE^–/–^ and non-agouti KKAy^–/–^ApoE^–/–^ mice

**FIGURE 1:**
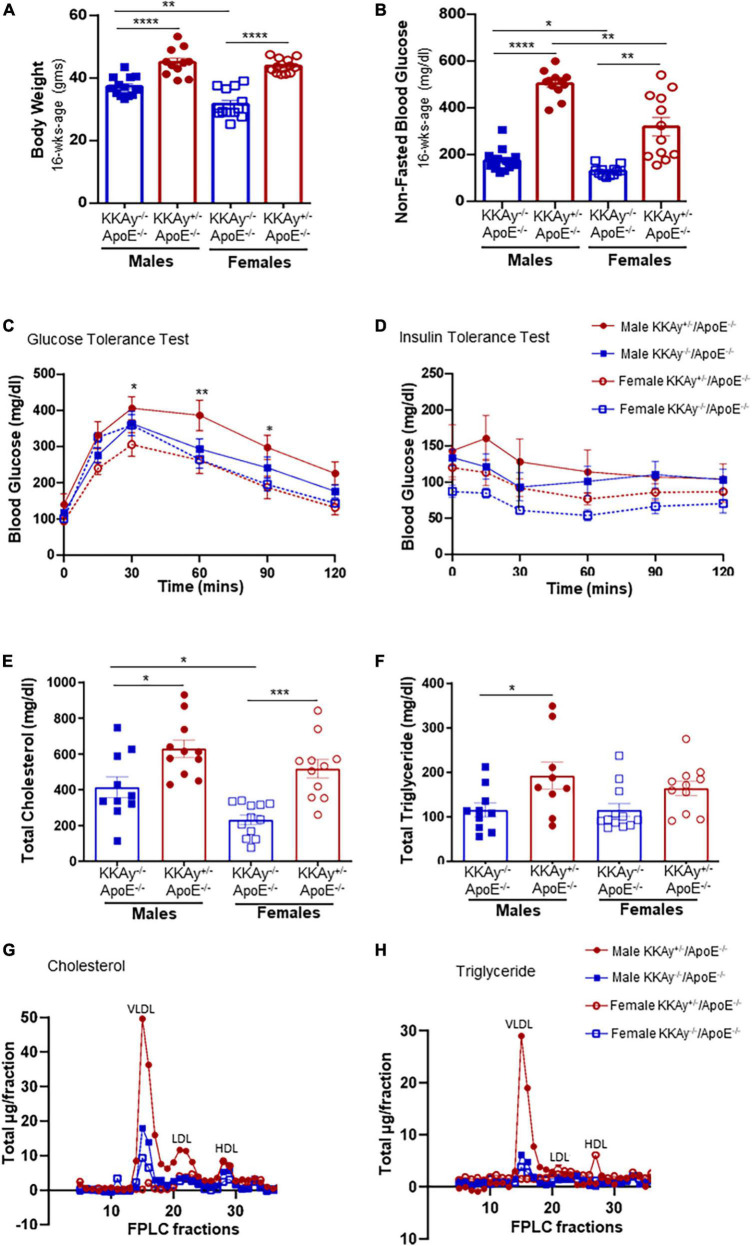
Sex-based differences in metabolic profile of agouti KKAy^+/–^ApoE^–/–^ and non-agouti KKAy^–/–^ApoE^–/–^ mice. Male and female age-matched KKAy^+/–^ApoE^–/–^ and KKAy^–/–^ApoE^–/–^ littermate mice were maintained on standard laboratory diet from 4 to 24 weeks of age. At 22 weeks of age, subsets of mice were subjected to either intraperitoneal glucose tolerance test (GTT) or insulin tolerance test (ITT) followed by mice harvest at 24 weeks age for blood and tissue collection. Shown are **(A)** body weight at 16 weeks age; **(B)** non-fasted blood glucose at 16 weeks age; **(C)** glucose tolerance test (GTT) *p < 0.05 and **p < 0.01 for male KKAy^+/–^ApoE^–/–^ vs. female KKAy^+/–^ApoE^–/–^; **(D)** insulin tolerance test (ITT); **(E)** plasma total cholesterol; **(F)** plasma total triglyceride; **(G)** cholesterol lipoprotein distribution via FPLC fractionation; **(H)** triglyceride lipoprotein distribution via FPLC fractionation. Results are expressed as mean ± SEM. For panels **(A,B,E,F)** ****p < 0.0001, ***p < 0.0005, **p < 0.005, *p < 0.05. *n* = 11–14/genotype/sex.

Yellow agouti KKAy^+/–^ApoE^–/–^ and age-matched black non-agouti KKAy^–/–^ApoE^–/–^ mice were monitored for body weight and non-fasted blood glucose levels once-a-month until harvest. Regardless of sex, body weight and non-fasted blood glucose levels were significantly elevated in 16-week-old agouti KKAy^+/–^ApoE^–/–^ mice compared with age-matched non-agouti KKAy^–/–^ApoE^–/–^ littermates (Figures 1A,B). Specifically, body weight was increased by 21 and 39% in male and female KKAy^+/–^ApoE^–/–^ vs. age- and sex-matched KKAy^–/–^ApoE^–/–^ littermate mice, respectively. Moreover, in non-agouti KKAy^–/–^ApoE^–/–^ genotypes, body weight was reduced by 15% in female vs male mice. Both male and female 16-week-old agouti KKAy^+/–^ApoE^–/–^ mice revealed non-fasted blood glucose levels >250 mg/dl, reflective of diabetes, with greater than 2-fold increase in glucose levels noted in these animals compared to age- and sex-matched non-agouti KKAy^–/–^ApoE^–/–^. In addition, a sex-specific difference in non-fasted blood glucose levels was observed in both agouti and non-agouti genotypes. Specifically, blood glucose was reduced by 25% in female non-agouti KKAy^–/–^ApoE^–/–^ mice compared to males, and in female agouti KKAy^+/–^ApoE^–/–^ mice it was reduced even further by 37% vs. male mice. Interestingly, GTT revealed a significant impairment in glucose tolerance in only male agouti KKAy^+/–^ApoE^–/–^ genotypes, while there was no difference in insulin sensitivity between the genotypes in either sex, shown via ITT (Figures 1C,D). We next measured plasma lipid levels in these animals. Regardless of sex, total cholesterol levels were significantly increased in both male and female agouti KKAy^+/–^ApoE^–/–^ mice as compared with age-matched KKAy^–/–^ApoE^–/–^ (Figure 1E). Specifically, there were 1.5- and 2.2-fold increases in plasma total cholesterol levels in male and female agouti KKAy^+/–^ApoE^–/–^ mice vs. corresponding non-agouti KKAy^–/–^ApoE^–/–^ genotypes, respectively. However, in non-agouti KKAy^–/–^ApoE^–/–^ mice, while plasma total cholesterol was 78% higher in male mice vs. age-matched females, no sex-specific difference was noted in the yellow agouti KKAy^+/–^ApoE^–/–^ genotype. Concomitant to total cholesterol levels, a significant increase in plasma total triglyceride was also noted in 16-week-old male agouti KKAy^+/–^ApoE^–/–^ mice (66% vs non-agouti KKAy^–/–^ApoE^–/–^ mice, *p* < 0.05; Figure 1F). However, there was no difference in plasma total triglycerides between the agouti and non-agouti genotypes in female mice. Likewise, no sex-specific difference in total triglyceride levels was observed in either the agouti or non-agouti genotypes. To further interrogate differences in plasma lipoprotein distribution, we next performed fast-performance liquid chromatography plasma fractionation. As shown in Figures 1G,H, only male agouti KKAy^+/–^ApoE^–/–^ mice revealed a significant increase in both VLDL-cholesterol and VLDL-triglyceride levels as compared to the other animal cohorts. Together, these results directly confirm the metabolic syndrome phenotype of yellow agouti KKAy^+/–^ApoE^–/–^ mice, characterized by increased body weight, blood glucose and total cholesterol levels. Moreover, our data demonstrate sex-specific differences in the metabolic profiles of agouti and non-agouti mice on an atherosclerotic background.

### Cardiometabolic fitness of agouti KKAy^+/–^ApoE^–/–^ and non-agouti KKAy^–/–^ApoE^–/–^ mice

**FIGURE 2:**
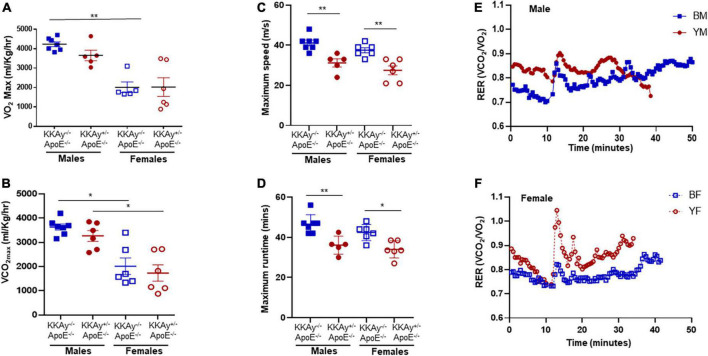
Sex-specific differences in cardiometabolic fitness of MetS KKAy^+/–^ApoE^–/–^ and non-MetS KKAy^–/–^ApoE^–/–^ mice using Treadmill Exercise Test. Mice were trained on the treadmill on day 1 and tested using a treadmill exercise test on day 2 with gradial increase in speed at constant incline. Shown are **(A)** maximum oxygen consumption (VO_2max_), **(B)** carbon dioxide produced relevant to maximal oxygen uptake (VCO_2max_), **(C)** maximum running speed, **(D)** maximum runtime until exhaustion. Line graphs depicting average RER for **(E)** representative male MetS KKAy^+/–^ApoE^–/–^ (YM) and non-MetS KKAy^–/–^ApoE^–/–^ (BM) mice and **(F)** representative female MetS KKAy^+/–^ApoE^–/–^ (YF) and non-MetS KKAy^–/–^ApoE^–/–^ (BF) mice. For panels **(A–D)**, data are mean ± SEM. For all the panels *n* = 5–7 mice per genotype. *p < 0.05, **p < 0.005.

To examine the cardiometabolic fitness of MetS and non-MetS atherosclerotic mice, a subset of male and female 23-week-old KKAy^+/–^ApoE^–/–^ and KKAy^–/–^ApoE^–/–^ mice were subjected to treadmill exercise test. Although a downward trend in maximum oxygen consumption (VO_2max_) was noted in MetS KKAy^+/–^ApoE^–/–^ mice during exercise as compared with non-MetS KKAy^–/–^ApoE^–/–^ genotypes of both sexes, this difference was not statistically significant (Figure 2A). Notably, while there was 2.1-fold decrease in VO_2max_ in female vs male mice among non-MetS KKAy^–/–^ApoE^–/–^ genotypes (*p* < 0.005), this sex-related difference was not observed in the MetS KKAy^+/–^ApoE^–/–^ mice. In addition, while we observed a sex-specific difference in VCO_2max_ in both MetS and non-MetS genotypes, there was no difference in VCO_2max_ between MetS and non-MetS animals (Figure 2B). Further, maximum run speed corresponding to maximal oxygen uptake and maximum runtime until exhaustion in MetS KKAy^+/–^ApoE^–/–^ mice were reduced by 24 and 20% respectively, vs non-MetS KKAy^–/–^ApoE^–/–^ genotypes, and this difference was noted in both sexes (*p* < 0.005; Figures 2C,D). RER, the ratio of carbon dioxide produced to oxygen consumed which can be used as an estimate of metabolic fuel usage, was consistently elevated in both male and female MetS KKAy^+/–^ApoE^–/–^ mice (Figures 2E,F). Together, these results suggest an impaired metabolic fitness in MetS KKAy^+/–^ApoE^–/–^ genotypes.

### Differences in lipid burden, plaque area and collagen content between MetS KKAy^+/–^ApoE^–/–^ and non-MetS KKAy^–/–^ApoE^–/–^ genotypes

**FIGURE 3:**
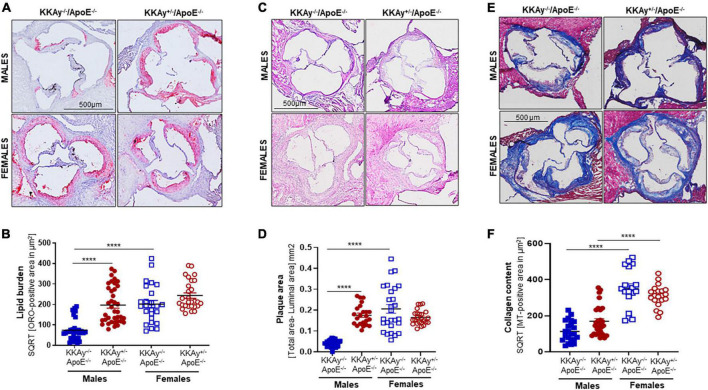
Sex-specific differences in lesion lipid burden, plaque area and collagen content in MetS KKAy^+/–^ApoE^–/–^ and non-MetS KKAy^–/–^ApoE^–/–^ mice. Serial sections of equivalent regions of the aortic root were stained with 0.5% Oil red O (ORO) followed by hematoxylin counterstaining. ORO-positive area (measured in μm^2^) was quantified using Image J. Shown are **(A)** representative ORO-stained aortic root sections, **(B)** summary data for ORO-positive area. *n* = 7–9 mice per genotype per sex (2–5 sections per mouse). Aortic root serial sections were stained with hematoxylin and eosin (H&E). Aortic root luminal area and total aortic root area (i.e., area enclosed by the internal elastic lamina in mm^2^) were measured using Image J; difference between total root area and luminal area denotes plaque area. Shown are **(C)** representative images for H&E-staining, **(D)** summary data for plaque area. *n* = 5–8 mice per genotype per sex (4–5 sections per mouse). Identical aortic root sections were stained with Masson’s Trichrome (MT) followed by hematoxylin counterstaining. MT-positive area (measured in μm^2^) depicting collagen content was quantified in Image J. Shown are **(E)** representative images for MT staining and **(F)** summary data for collagen content, *n* > 12 individual sections for each genotype per sex. All images were captured at 10× magnification. ****p < 0.0001.

Using aortic root morphometry, we next examined atherosclerotic lesion formation in 24-week-old MetS KKAy^+/–^ApoE^–/–^ and age-matched non-MetS KKAy^–/–^ApoE^–/–^ genotypes. Oil red O (ORO) staining of aortic root sections revealed robust lipid-filled lesions in MetS KKAy^+/–^ApoE^–/–^ mice of both sexes and in female non-MetS KKAy^–/–^ApoE^–/–^ mice (Figures 3A,B). Specifically, in male mice, aortic root lipid burden was increased by 5.7-fold in KKAy^+/–^ApoE^–/–^ as compared with non-MetS KKAy^–/–^ApoE^–/–^ mice (*p* < 0.0001). In contrast, no statistically significant difference in lipid burden was noted between MetS and non-MetS genotypes in the female mice. Of note, ORO-positive staining area, reflective of lipid burden, was significantly higher in the female non-agouti KKAy^–/–^ApoE^–/–^ mice (6-fold vs. male KKAy^–/–^ApoE^–/–^; *p* < 0.0001). Next, we assessed the plaque area using H&E staining of aortic root sections. Consistent with the enhanced ORO-positive lipid burden, we found a significant increase in plaque area in MetS KKAy^+/–^ApoE^–/–^ aortic root sections compared with those derived from non-MetS KKAy^–/–^ApoE^–/–^ mice. Moreover, this difference in plaque area was specific to only male mice (Figures 3C,D, *p* < 0.0001), with no statistically significant difference between the female genotypes. Of note, elevated lipid burden in root sections of female black KKAy^–/–^ApoE^–/–^ mice was accompanied with increased plaque area in these animals. Interestingly, Masson’s Trichrome (MT) staining of aortic root sections did not reveal a statistically significant difference in lesion collagen content between MetS KKAy^+/–^ApoE^–/–^ and non-MetS KKAy^–/–^ApoE^–/–^ genotypes, regardless of sex. However, both agouti and non-agouti female mice revealed a much higher collagen content in aortic root lesions as compared to the corresponding male genotypes. Specifically, MT-positive collagen content was profoundly augmented in female vs. male mice in both genotypes (Figures 3E,F, KKAy^–/–^ApoE^–/–^: 3-fold; KKAy^+/–^ApoE^–/–^: 1.8-fold). Together, these results clearly demonstrate sex-specific differences in atherogenic phenotype between agouti MetS and non-agouti non-MetS mice on an atherosclerotic background.

### Sex-specific differences in inflammatory and SMC lesion burden between MetS KKAy^+/–^ApoE^–/–^ and non-MetS KKAy^–/–^ApoE^–/–^ mice

**FIGURE 4:**
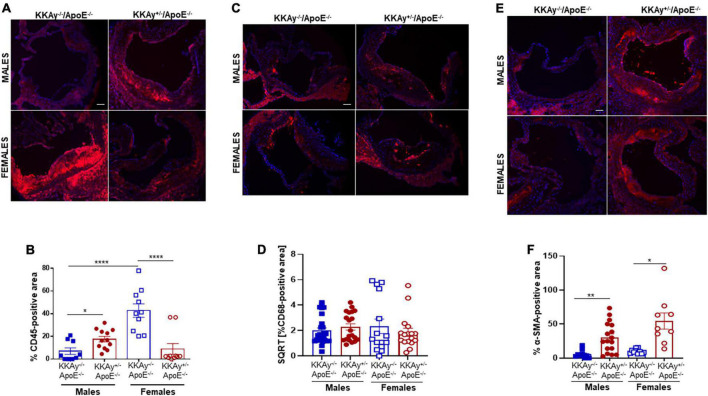
Leukocyte and macrophage infiltration and SMC content in aortic root lesions of MetS KKAy^+/–^ApoE^–/–^ and non-MetS KKAy^–/–^ApoE^–/–^ mice. Aortic root sections were subjected to immunohistochemistry using CD45, CD68, and α-SMA antibodies. Images were taken at 10x magnification. Total lesion area and positive staining area of lesions (in sq. pixels) were quantified in each section using Image J. Shown are **(A)** representative images for CD45 staining (leukocyte content), **(B)** summary graph for %CD45-positive area, **(C)** representative images for CD68 staining (macrophage content), **(D)** summary graph for %CD68-positive area, **(E)** representative images for α-SMA staining (SMC content), and **(F)** summary graph for %SMA-positive area (*n* = 5–6 mice per genotype per sex (2–4 sections per mouse). All values are expressed as mean ± SEM; *p < 0.05, **p < 0.005, ****p < 0.0001, scale bar = 115 μm.

Immunohistochemistry demonstrated enhanced CD45 expression, reflective of leukocyte infiltration, in aortic root lesions of MetS KKAy^+/–^ApoE^–/–^ vs. age-matched non-MetS KKAy^–/–^ApoE^–/–^ genotypes in male mice. In contrast, CD45 expression was significantly lower in aortic root lesions of MetS agouti vs. non-MetS non-agouti female mice (Figure 4A). Specifically, while male KKAy^+/–^ApoE^–/–^ demonstrated 2.5-fold increase in CD45 expression (*p* < 0.05 vs. male KKAy^–/–^ApoE^–/–^), CD45-stained positive area was reduced by 79% in MetS KKAy^+/–^ApoE^–/–^ vs. non-MetS KKAy^–/–^ApoE^–/–^ genotypes in female mice (Figure 4B, *p* < 0.0001). Interestingly, the highest leukocyte burden was detected in aortic root lesions of non-MetS KKAy^–/–^ApoE^–/–^ females; specifically, CD45 expression increased by 6-fold (*p* < 0.0001) in these animals compared to the male genotypes. We next assessed the macrophage content of lesions in these animals via CD68 immunostaining. As shown in Figures 4C,D, the difference in CD68 expression depicting macrophage infiltration in aortic root lesion of KKAy^+/–^ApoE^–/–^ vs. KKAy^–/–^ApoE^–/–^ failed to reach statistical significance, and this effect was consistent in both male and female mice. These data suggest a lack of sex-specific difference in macrophage infiltration between MetS and non-MetS genotypes. To define SMC distribution in lesions, we next performed α-SMA immunostaining of aortic root sections. As shown in Figure 4E, there was a significant increase in α-SMA expression, illustrating augmented lesion SMC content in agouti KKAy^+/–^ApoE^–/–^ vs. age-matched non-agouti KKAy^–/–^ApoE^–/–^ mice. Moreover, this increase in SMC abundance in the lesions was noted in both male and female mice, with > 5-fold increase in MetS vs. non-MetS genotypes (Figure 4F, *p* < 0.005 in males; *p* < 0.05 in females). Together, these results clearly demonstrate sex-specific differences in lesion inflammatory and SMC content between MetS and non-MetS mice on an atherosclerotic background.

### Increased TSP-1 expression associates with reduced LMOD-1 and SRF expression specific to male mice

**FIGURE 5:**
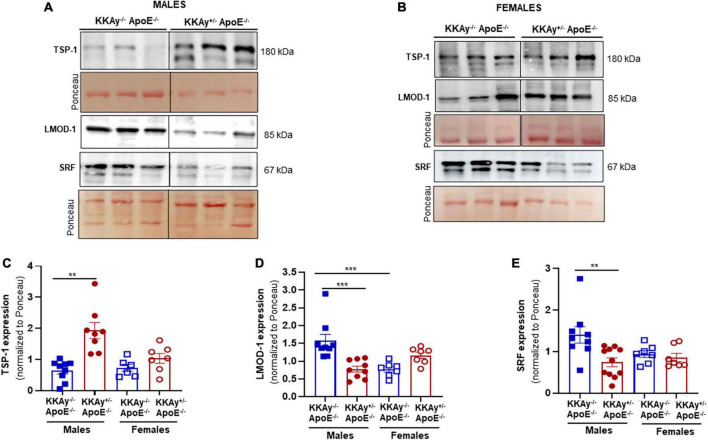
Increased TSP-1 expression is accompanied with reduced LMOD-1 and SRF expression in MetS KKAy^+/–^ApoE^–/–^ mice. Shown are **(A)** representative western blots for TSP-1, LMOD-1, and SRF expression in aortic lysates from male MetS KKAy^+/–^ApoE^–/–^ and non-MetS KKAy^–/–^ApoE^–/–^ mice; **(B)** representative western blots for TSP-1, LMOD-1, and SRF expression in aortic lysates from female MetS KKAy^+/–^ApoE^–/–^ and non-MetS KKAy^–/–^ApoE^–/–^ mice. Shown are summary graphs depicting densitometric quantification of western blots for panels **(C)** TSP-1; **(D)** LMOD-1, and **(E)** SRF expression (n = 6–11 animals per genotype per sex). Loading control = Ponceau S. Data shown illustrates protein expression normalized to Ponceau S. Results are expressed as mean ± SEM. **p < 0.005; ***p < 0.0005. For each representative immunoblot, lane images depict proteins loaded and detected on a single immunoblot (full blots shown in Supplementary Figures 1, 2); however, lanes were re-arranged to improve the clarity of presentation.

Concomitant to increased atherosclerotic lesion burden in male MetS KKAy^+/–^ApoE^–/–^ mice, immunoblotting of aortic tissue lysates revealed augmented TSP-1 expression in these animals. Specifically, TSP-1 protein expression increased by 3-fold in aortic vessels of agouti KKAy^+/–^ApoE^–/–^ male mice (*p* = 0.0056 vs. non-agouti KKAy^–/–^ApoE^–/–^ males). This increase in TSP-1 expression was accompanied with reduced LMOD-1 and SRF expression in male KKAy^+/–^ApoE^–/–^ genotypes (50 and 55%, respectively, vs. KKAy^–/–^ApoE^–/–^ male mice, *p* < 0.005; Figures 5A,C–E). On the contrary, there was no statistically significant difference in TSP-1, LMOD-1, and SRF expression between MetS and non-MetS genotypes in female mice (Figures 5B,C–E). These data clearly demonstrate a correlation between augmented TSP-1 expression and reduced LMOD-1 and SRF expression in MetS KKAy^+/–^ApoE^–/–^ genotypes, that is specific to only male mice.

### Lack of TSP-1 upregulates SM contractile marker expression specific to male mice

**FIGURE 6:**
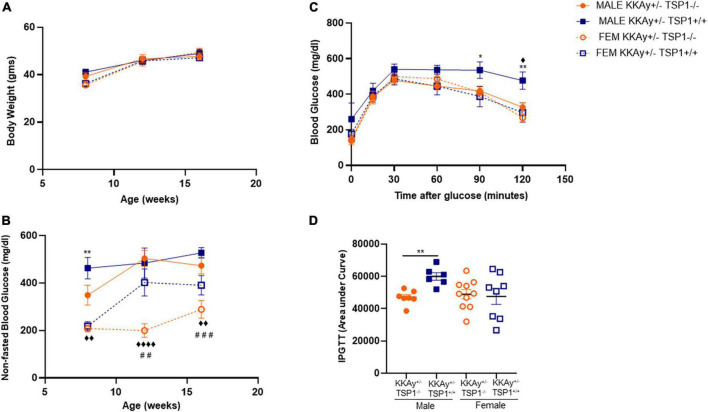
Effect of global TSP-1 deletion on metabolic profile of MetS KKAy^+/–^ mice. Male and female age-matched agouti KKAy^+/–^TSP-1^+/+^ and KKAy^+/–^TSP-1^–/–^ littermate mice were maintained on standard lab diet from 4 to 18 weeks of age. Shown are **(A)** body weight and **(B)** non-fasted blood glucose levels measured in subsets of mice genotypes of each sex at 8-, 12- and 16-weeks-age. ^◆^*p* < 0.005, ^◆◆◆◆^*p* < 0.0001 denotes male KKAy^+/–^TSP-1^–/–^ vs. female KKAy^+/–^TSP-1^–/–^; **p < 0.005 denotes male KKAy^+/–^TSP-1^+/+^ vs. female KKAy^+/–^TSP-1^+/+^; ^##^*p* < 0.005, ^###^*p* < 0.0005 denotes female KKAy^+/–^TSP-1^+/+^ vs. female KKAy^+/–^TSP-1^–/–^. **(C)** glucose tolerance test (GTT) done at 17 weeks age. *p < 0.05, **p < 0.005 denotes male KKAy^+/–^TSP-1^+/+^ vs. female KKAy^+/–^TSP-1^+/+^, ^◆^*p* < 0.05 denotes male KKAy^+/–^TSP-1^+/+^ vs. male KKAy^+/–^TSP-1^–/–^. **(D)** Area under curve for GTT. *n* = 7–10 per genotype per sex for panels **(B,C)**; *n* = 5–10 per genotype per sex for panels **(D,E)**. All results are expressed as mean ± SEM.

**FIGURE 7:**
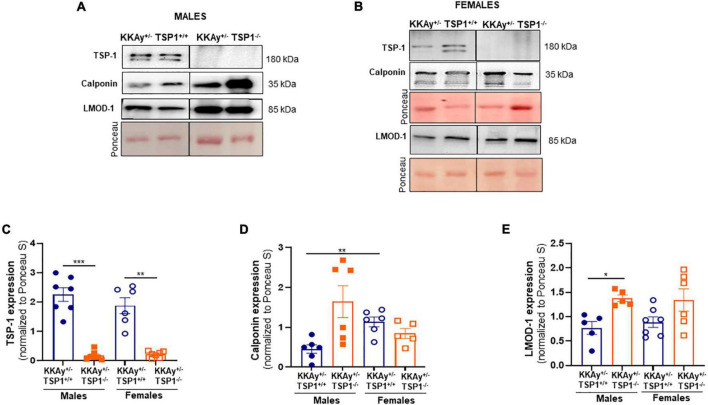
TSP-1 deletion increases LMOD-1 and calponin expression in aortic vessels of male MetS KKAy^+/–^ mice. Shown are representative western blots for TSP-1, LMOD-1 and calponin expression in aortic lysates derived from **(A)** male KKAy^+/–^TSP-1^+/+^ and KKAy^+/–^TSP-1^–/–^ mice and **(B)** female KKAy^+/–^TSP-1^+/+^ and KKAy^+/–^TSP-1^–/–^ mice. Shown are summary graphs for densitometric quantification of immunoblots for **(C)** TSP-1, **(D)** Calponin, and **(E)** LMOD-1 expression. *n* = 5–7 animals per genotype per sex. All results are expressed as mean ± SEM. *p < 0.05, ***p < 0.0005. Loading control = Ponceau S. Data shown illustrates protein expression normalized to Ponceau S. For representative immunoblots, lane images depict proteins loaded and detected on a single immunoblot (full blots shown in Supplementary Figures 3, 4); however, lanes were re-arranged to improve the clarity of presentation.

To interrogate whether TSP-1 plays a direct role in SMC de-differentiation in MetS, we generated MetS KKAy^+/–^ mice with and without global TSP-1 deletion. Metabolic profiling of these animals revealed sex-specific differences in blood glucose and glucose tolerance between agouti KKAy^+/–^TSP-1^+/+^ and KKAy^+/–^TSP-1^–/–^ mice, without significant changes in body weight (Figure 6). Specifically, in mice with intact TSP-1, non-fasted blood glucose was lower in the female genotypes at 8 weeks of age (*p* < 0.005); however, this sex-specific difference in blood glucose level was obviated at later time points. Lack of TSP-1 significantly reduced non-fasted blood glucose levels in female agouti KKAy^+/–^ vs. male agouti KKAy^+/–^ genotypes as early as 8 week of age, and this effect was sustained until 16 weeks age (*p* < 0.005). Moreover, KKAy^+/–^TSP-1^–/–^ mice exhibited a significant decrease in non-fasted blood glucose vs. KKAy^+/–^TSP-1^+/+^ only in the female genotypes. In contrast, TSP-1 deletion had no effect on blood glucose levels in male agouti genotypes (Figure 6B). GTT further revealed that in the presence of intact TSP-1, male KKAy^+/–^ were significantly glucose intolerant compared with female genotypes at both 90- and 120-min after glucose injection. In addition, KKAy^+/–^ mice lacking TSP-1 manifested impaired glucose tolerance 120-min following glucose injection vs. KKAy^+/–^ with intact TSP-1 in male mice; however, this difference in glucose tolerance was not observed in female agouti genotypes with and without TSP-1 (Figures 6C,D). Immunoblotting of aortic lysates demonstrated that TSP-1 deletion augmented LMOD-1 expression (SM contractile marker) in aortic vessels of male MetS KKAy^+/–^ mice (Figures 7A,C,E). Specifically, there was >1.7-fold increase in LMOD-1 expression (Figures 7A,E; *p* < 0.05) in male KKAy^+/–^TSP-1^–/–^ aortic vessels vs. age-matched KKAy^+/–^TSP1^+/+^ mice (with intact TSP-1). While an upward trend was also noted in calponin expression in aortic vessels derived from male agouti KKAy^+/–^ mice lacking TSP-1, this increase was not statistically significant (Figures 7A,D). On the contrary, TSP-1 loss had no effect on SM contractile marker expression in aortic vessels derived from MetS KKAy^+/–^TSP1^–/–^ vs. agouti genotypes with intact TSP-1 in female mice (Figures 7B,D,E). Moreover, while Calponin expression significantly differed between male and female KKAy^+/–^ aortic vessels with intact TSP-1, sex-specific difference noted among the TSP-1 knockouts failed to reach statistical significance. Together, these data suggest a regulatory role of TSP-1 on SMC de-differentiation, characterized by reduced SM contractile marker expression, in MetS specific to male genotypes.

### Sex-specific differences in plasma testosterone levels in MetS KKAy^+/–^ApoE^–/–^ and non-MetS KKAy^–/–^ApoE^–/–^ mice

**FIGURE 8:**
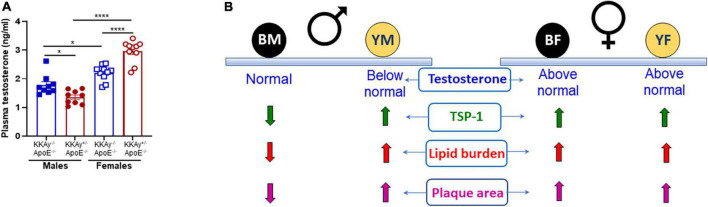
Sex-specific differences in plasma testosterone levels in MetS KKAy^+/–^ApoE^–/–^ and non-MetS KKAy^–/–^ApoE^–/–^ mice. **(A)** Shown are summary graphs for plasma testosterone levels in 24-weeks-old male and female MetS KKAy^+/–^ApoE^–/–^ and non-MetS KKAy^–/–^ApoE^–/–^ mice. *p < 0.05; ****p < 0.0001 (*n* = 9–10 mice per genotype per sex). **(B)** Overview of findings. BM, black male KKAy^–/–^ApoE^–/–^; YM, yellow male KKAy^+/–^ApoE^–/–^; BF, black female KKAy^–/–^ApoE^–/–^; YF, yellow female KKAy^+/–^ApoE^–/–^.

To interrogate the link between sex hormones and atherogenic phenotype observed in MetS vs non-MetS mice, we measured plasma testosterone levels in a subset of our animals. As shown in Figure 8A, testosterone levels were significantly elevated in both MetS and non-MetS genotypes in female mice as compared to the corresponding male genotypes. Specifically, there was 20% increase in testosterone concentration in female non-agouti KKAy^–/–^ApoE^–/–^ vs. age-matched male non-agouti genotypes. In case of MetS KKAy^+/–^ApoE^–/–^ mice, testosterone levels increased >2-fold in female vs male mice. Interestingly, both agouti and non-agouti female mice displayed remarkably higher testosterone concentrations compared to levels typically reported in healthy females (33). In contrast, testosterone levels were found to be considerably lower in male agouti KKAy^+/–^ApoE^–/–^ mice compared to normal values reported in healthy males (33). Furthermore, while in female mice, testosterone levels were augmented in MetS KKAy^+/–^ApoE^–/–^ (76% vs. non-MetS KKAy^–/–^ApoE^–/–^; *p* < 0.0001), male MetS genotypes revealed attenuated testosterone levels vs. non-MetS mice. Collectively, these data clearly demonstrate sex-related differences in plasma testosterone levels in MetS and non-MetS genotypes.

## Conclusion

In the current study, we provide novel evidence for sex-specific differences in atherosclerotic lesion burden, expression of SM contractile markers and a proatherogenic protein TSP-1 expression between MetS and non-MetS mice. Our results suggest a link between accelerated atherosclerosis and TSP-1 expression in the vessel wall, specific to male MetS mice. Notably, our data implies a sex-specific causal role of TSP-1 on SMC de-differentiation in MetS. It is a generally accepted dogma that men and women present significant differences in the incidence and progression of atherosclerosis and related cardiovascular complications. Yet, the molecular basis of such sexual dimorphism has remained inconclusive, to a large extent, due to the lack of robust preclinical animal studies employing both sexes.

The KKAy^+/–^ mouse is a classical murine model of MetS characterized by hyperglycemia, insulin resistance, glucose intolerance and severe hyperinsulinemia by approximately 8 weeks of age. Previous studies have reported (31) that KKAy^+/–^ mice develop polygenic forms of metabolic anomalies, analogous to humans. Accordingly, in the present work, we chose the KKAy^+/–^ mice backcrossed for six generations with ApoE^–/–^ to generate a mouse model of combined MetS and atherosclerosis. In line with earlier findings (34), our data confirm the MetS phenotype of agouti KKAy^+/–^ApoE^–/–^ mice, characterized by hyperglycemia, obesity, and dyslipidemia, as compared with non-agouti KKAy^–/–^ApoE^–/–^ littermates. Of note, only male agouti KKAy^+/–^ApoE^–/–^ were observed to be glucose intolerant and hypertriglyceridemic versus age-matched non-agouti KKAy^–/–^ApoE^–/–^ males. Interestingly, a remarkable sex-specific difference in plasma total cholesterol was noted only in the non-agouti genotypes, with male KKAy^–/–^ApoE^–/–^ revealing elevated total cholesterol levels vs. age-matched female genotypes. The observed differences between the agouti and non-agouti mice in the current study emphasize the need for using age-matched littermate mice for such studies. Moreover, our data suggest that knockout of ApoE in female non-agouti KKAy^–/–^ genotypes may trigger a state of hypertriglyceridemia in these animals. Consistent with earlier reports (34), lack of ApoE in agouti KKAy^+/–^ mice led to significant aberrations in the lipoprotein distribution patterns in these animals, with elevated VLDL-cholesterol and VLDL-triglyceride levels. However, this difference was specific to only male genotypes implicating sex-related differences in this murine model of combined MetS and atherosclerosis. Owing to limited plasma sample volume availability, we were unable to measure plasma insulin levels in our animals. However, based on earlier published literature (35, 36), we expect the agouti KKAy^+/–^ApoE^–/–^ mice to have elevated insulin levels compared to non-agouti genotypes. Indeed, previous studies have reported increased pancreatic beta cell degranulation and islet cell hypertrophy triggering increased insulin production in KKAy^+/–^ mice, and such observations were noted as early as 5-weeks of age.

Metabolic profiling of MetS agouti KKAy^+/–^ApoE^–/–^ mice revealed sex-specific differences in response to forced activity. Maximal oxygen consumption was reduced in female non-MetS KKAy^–/–^ApoE^–/–^ mice compared to male mice, and this sex-specific phenotype was lost in MetS KKAy^+/–^ApoE^–/–^ genotypes. In both male and female mice, run time until exhaustion was significantly lower in the MetS genotypes, suggesting lower exercise performance in these animals, compared with non-MetS genotypes; these observations are consistent with what is expected in obese mice. RER, which can be used to estimate metabolic fuel oxidation, was elevated in both male and female MetS KKAy^+/–^ApoE^–/–^ mice, indicating potentially increased glucose oxidation over fat (37) in these mice.

Among the currently available mouse models of diabetes and obesity, ob/ob and db/db mice are the most extensively utilized murine models in cardiovascular research. Despite relative similarities in the metabolic phenotype of ob/ob and db/db mice (38), previous studies have reported considerable variability in the extent of atherosclerotic lesions that develop in these mice on an ApoE^–/–^ background. For instance, obese hyperglycemic leptin-deficient ob/ob;ApoE^–/–^ mice have been reported to develop relatively smaller lesions, with marked suppression in lesion progression from fatty streaks to fibrous plaques as compared to ApoE^–/–^ mice (39). On the other hand, leptin receptor-deficient db/db;ApoE^–/–^ have demonstrated accelerated atherosclerosis accompanying hyperglycemia, obesity, hyperinsulinemia and dyslipidemia vs. age-matched ApoE^–/–^ mice (40). It should be noted that while metabolic anomalies in ob/ob and db/db mice are consistent with that observed in humans, leptin-deficiency or leptin receptor mutation are rare or absent in individuals with MetS. To this end, the agouti KKAy^+/–^ApoE^–/–^ mice developed in this study more closely mimics the clinical features associated with MetS.

While both male and female MetS KKAy^+/–^ApoE^–/–^ mice developed profound lipid-filled lesions accompanying increased plaque area, there was a significant difference in the progression of atherosclerotic lesions between MetS and non-MetS genotypes that was specific to only male mice. While these results are in line with a previous report (34), the current work has provided the first evidence of sex-specific variations in lesion development in this murine model of MetS. Moreover, atherosclerotic lesion formation correlated with augmented TSP-1 expression in the vascular wall. TSP-1 is a multifunctional protein with cell- and tissue-specific effects, attributed to its differential interaction with and regulation of several cell-surface receptors and matrix-binding partners, in turn modulating cell-cell and cell-matrix interactions (41). Accumulating literature indicates that TSP-1 signaling via differential receptor activation (CD36, CD47) regulates the function of different vascular cell types, triggering inflammatory cell adhesion and transmigration through the vascular endothelium, LDL pinocytosis by macrophages and foam cell formation, endothelial cell (EC) dysfunction and EC senescence (17, 42, 43). In a healthy vessel, TSP-1 expression is typically low; however, in response to proatherogenic insults such as hyperglycemia and obesity, TSP-1 expression is significantly augmented. Previous studies have shown increased TSP-1 expression in injured vessels and atherosclerotic lesions (23, 25). *In vitro* findings from our laboratory demonstrate that both high glucose and high leptin, at concentrations reflective of diabetes and obesity respectively, stimulate TSP-1 expression in human aortic SMC primary cultures (15, 16). We previously reported that global TSP-1 loss attenuates lesion burden and proliferative VSMC content in STZ-induced hyperglycemic ApoE^–/–^ mice (26). In a follow-up study, we further showed that global TSP-1 deletion abrogates lipid-filled lesion burden and VSMC abundance in ApoE^–/–^ mice treated with recombinant leptin, at concentrations mimicking obesity (27). In the context of the present study, our findings that augmented TSP-1 expression in the vessel wall associates with robust atherosclerotic lesions displaying increased SMC content in MetS KKAy^+/–^ApoE^–/–^ mice underscore a TSP-1-dependent mechanism underlying MetS-induced atherosclerosis.

Clinical and animal studies have highlighted a role of TSP-1 in the pathophysiology of metabolic disorders including diabetes and obesity (17). For instance, Li et al. (44) reported improved glucose tolerance and increased insulin sensitivity in obese TSP-1 knockout mice vs. obese wild-type mice in response to high fat feeding. Such improvement in metabolic profile was attributed to reduced systemic and adipose tissue inflammation in association with reduced macrophage accumulation in the adipose tissue of these animals. Likewise, a subsequent study demonstrated that myeloid-specific TSP-1 deletion protects long term diet-induced obese mice against inflammation and insulin resistance (45). In line with these earlier reports, we have shown that TSP-1 deletion in MetS agouti KKAy^+/–^ mice significantly improved glucose tolerance in these animals compared with MetS genotypes with intact TSP-1. Of particular note, the improved metabolic profile in these animals was found to be specific to only male genotypes. These data suggest a sex-related differential role of TSP-1 on glucose metabolism and support a therapeutic potential of TSP-1 in metabolic disease.

In murine models of vascular injury, TSP-1 has been reported to facilitate arterial SMC activation (46, 47), attributing to its proatherogenic properties. Earlier findings from our laboratory indicate that under high glucose- or high leptin-stimulated conditions, augmented TSP-1 expression associates with increased VSMC proliferation (15, 16, 48). Conversely, we have shown that loss of TSP-1 attenuated VSMC proliferative response to high glucose or high leptin *in vitro* (15, 16). In line with these findings, we further reported that global TSP-1 deficiency blocks proliferative SMC lesion abundance in both STZ-induced hyperglycemic and leptin-treated ApoE^–/–^ mice (26, 27). In view of this literature, our data raises the possibility that TSP-1 drives SMC de-differentiation in MetS in a sex-specific manner. Future studies are currently underway in our laboratory to interrogate whether TSP-1 directly modulates VSMC fate changes to a diseased phenotype in MetS, and to delineate underlying molecular mechanisms. Additionally, our findings suggest a sex-specific interaction of TSP-1 with lesion inflammatory burden in MetS KKAy^+/–^ApoE^–/–^ mice. Specifically, while lesion leukocyte infiltration was increased in male agouti KKAy^+/–^ApoE^–/–^ concomitant to enhanced TSP-1 expression in the vascular wall, inflammatory cell content was markedly attenuated in MetS KKAy^+/–^ApoE^–/–^ vs. non-MetS genotypes in female mice. These data illuminate sex-specific differences in lesion inflammatory status driven by MetS, highlighting the need for sex-based precision medicine to combat MetS-induced vascular dysfunction.

Accumulating epidemiological studies have suggested that men have a higher propensity for atherosclerotic disease than women, particularly at a younger age. However, as opposed to clinical data, animal studies on sex differences in plaque size and plaque burden have yielded conflicting results. For example, while some reports (49, 50) have suggested an atheroprotective effect of higher estrogen levels in female mice, others, summarized in (28) have shown either enhanced or similar plaque area in female ApoE^–/–^ vs. age-matched male mice. In agreement with these earlier reports, we show augmented atherogenic phenotypes in both male and female MetS mice on ApoE^–/–^ background. It would be remiss not to mention that in the current study, female non-agouti KKAy^–/–^ApoE^–/–^ mice developed large lipid-filled lesions resulting in enhanced plaque area within aortic roots similar to lesion progression in age-matched female agouti KKAy^+/–^ApoE^–/–^ mice; moreover, increased plaque area and lipid burden was associated with elevated TSP-1 expression in both genotypes in the female mice. Emerging literature support the idea that sex hormones (testosterone and estrogen) and sex chromosomes as well as their interactive pathways may contribute to sex-specific differential effects in various pathological conditions. Testosterone and estrogen are known to affect atherosclerosis in both sexes and multiple mechanisms have been postulated to play an underlying role (50). While testosterone was previously reported to improve insulin sensitivity, lower visceral adiposity and exert vasodilatory effects (51), clinical and animal research on the role of testosterone in atherosclerosis and related cardiovascular health remains inconclusive and rather contradictory (52, 53). For instance, multiple epidemiological studies suggest that testosterone deficiency may attribute to an increased incidence of adverse cardiovascular events (54–56). Congruently, animal studies using atherosclerotic murine models have shown that testosterone can retard atherogenesis (57, 58), possibly via its conversion to cardioprotective estradiol in the vessel wall. Few other studies have reported a beneficial effect of short- or long-term testosterone administration on arterial stiffness and vasomotor function (52). In the current work, we found significant differences in plasma testosterone levels between MetS and non-MetS genotypes, and this difference was observed in both male and female mice. Of note, while testosterone levels in both MetS and non-MetS female mice were found to be considerably above the typical range reported for healthy females (33, 59), only male MetS genotypes revealed plasma testosterone relatively below values typically reported for healthy males (33, 60, 61). Intriguingly, in the current study we found that mice with either above normal or below normal testosterone concentrations developed robust lipid-filled lesions. Accordingly, we speculate that supraphysiological testosterone levels may contribute to worsened atherosclerosis in MetS and non-MetS female genotypes compared to male KKAy^–/–^ mice. Paradoxically, it is likely that testosterone deficiency in male agouti KKAy^+/–^ApoE^–/–^ may be responsible for accelerated atherosclerosis vs. non-agouti genotypes. These data lend additional support to the concept that testosterone concentrations both above, and below physiologic range may increase risks of atherosclerotic disease. In terms of the relationship between testosterone and TSP-1, our data prompt us to speculate that low testosterone concentrations as noted in male MetS genotypes may induce TSP-1 expression in the vessel wall, thereby blocking SMC differentiation. Additional studies to delineate the underlying molecular mechanism are warranted.

The current study has a few limitations. A limitation of our study design is that ApoE^–/–^ mice lacking the diabetic KK background was not utilized. We postulate that the diabetic background is necessary to facilitate atherosclerosis such that in the absence of the KK background, animals may fail to show detectable lesions at the timepoint studied. Thus, while the KK background alone may result in a pre-diabetic phenotype manifesting sex-specific differences in lesion burden and proatherogenic status, an ectopic expression of the agouti gene on the KK background (KKAy^+/–^) is more likely to augment TSP-1 expression accelerating atherosclerosis, and this effect occurs independent of differences in sex. It should be further noted that while our study suggests a causal role of TSP-1 in atherosclerosis driven by metabolic aberrations, whether TSP-1 plays a direct role in MetS-induced atherosclerosis is yet to be determined and would require generation of atherosclerotic KKAy^+/–^ApoE^–/–^ mice lacking TSP-1. While such studies are beyond the scope of this manuscript, future work in our laboratory will interrogate the contribution of VSMC-derived TSP-1 to atherosclerotic lesion progression and VSMC phenotypic transformation prompted by MetS, using VSMC-specific TSP-1 knockout mice expressing VSMC-specific reporter gene to enable lineage tracing. Finally, although we did not measure plasma estradiol in the current study due to limited sample availability, we do acknowledge its role in vascular pathology. Future studies are warranted to determine the relative contribution of testosterone vs. estradiol to MetS-induced atherosclerosis and delineate underlying molecular mechanisms.

In conclusion, the present study provides the first demonstration of sex-specific differences in the development of atherosclerosis and TSP-1 expression in MetS vs non-MetS mice. Our data suggest a sex-specific differential role of TSP-1 on lesion progression and SMC differentiation in MetS. These findings underscore a fundamental link between TSP-1 and VSMC phenotypic switching in MetS. Importantly, the current work provides evidence for a role of testosterone prompting sex-related differences in lesion severity and complexity (Figure 8B), independent of MetS.

## Data availability statement

The original contributions presented in this study are included in the article/Supplementary material, further inquiries can be directed to the corresponding author.

## Ethics statement

The animal study was reviewed and approved by Northeast Ohio Medical University IACUC.

## Author contributions

SG designed and performed the experiments, analyzed the data, and wrote the first draft of the manuscript. SK and NB performed the experiments and analyzed the data. AM and JL performed the mice genotyping, measured body weight and blood glucose, and sectioned aortic root samples. AK performed the imaging data analyses. JF assisted with treadmill exercise test and reviewed and revised the manuscript. PR conceived the project, designed the experiments, analyzed the data, and revised and edited the manuscript. All authors contributed to the article and approved the submitted version.
