# Supplementary figures and images for: Sex-specific differences in atherosclerosis, thrombospondin-1, and smooth muscle cell differentiation in metabolic syndrome versus non-metabolic syndrome mice
Source: Front Cardiovasc Med. 2022 Nov 23;9:1020006. doi: 10.3389/fcvm.2022.1020006 (PMC9727198; doi:10.3389/fcvm.2022.1020006)

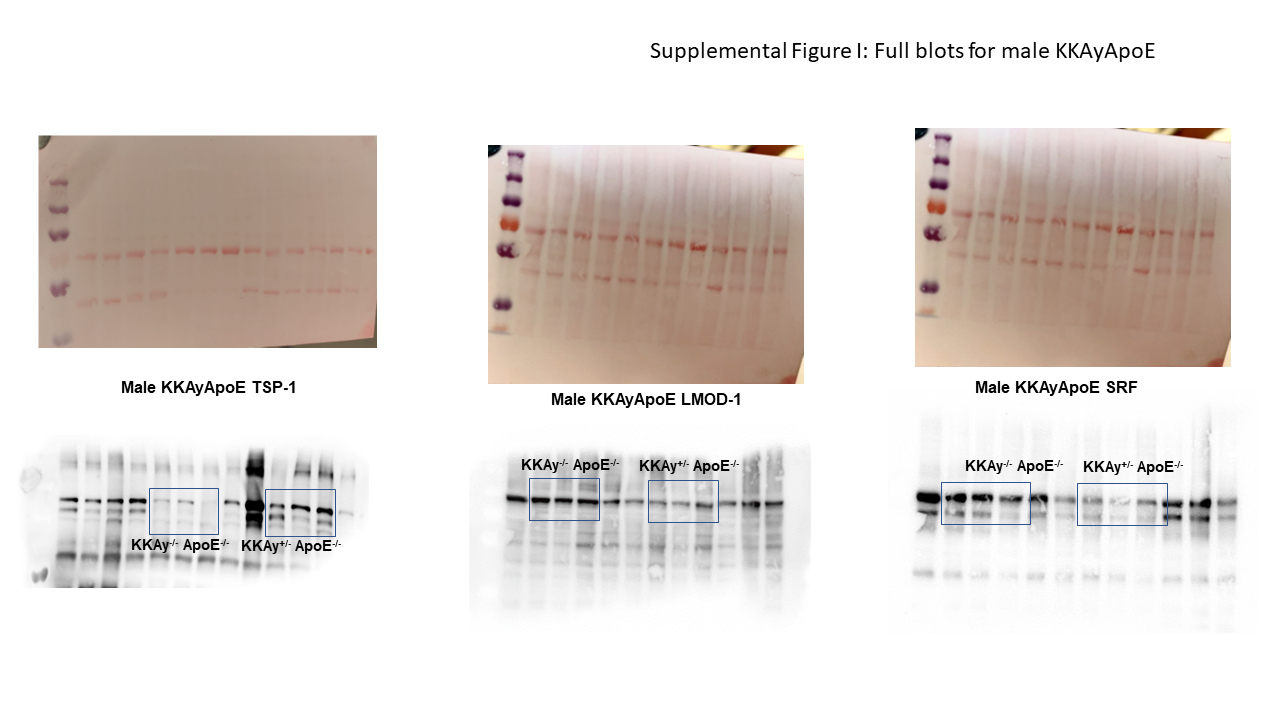

Supplement: Supplementary file 1 [file Image_1.tif]

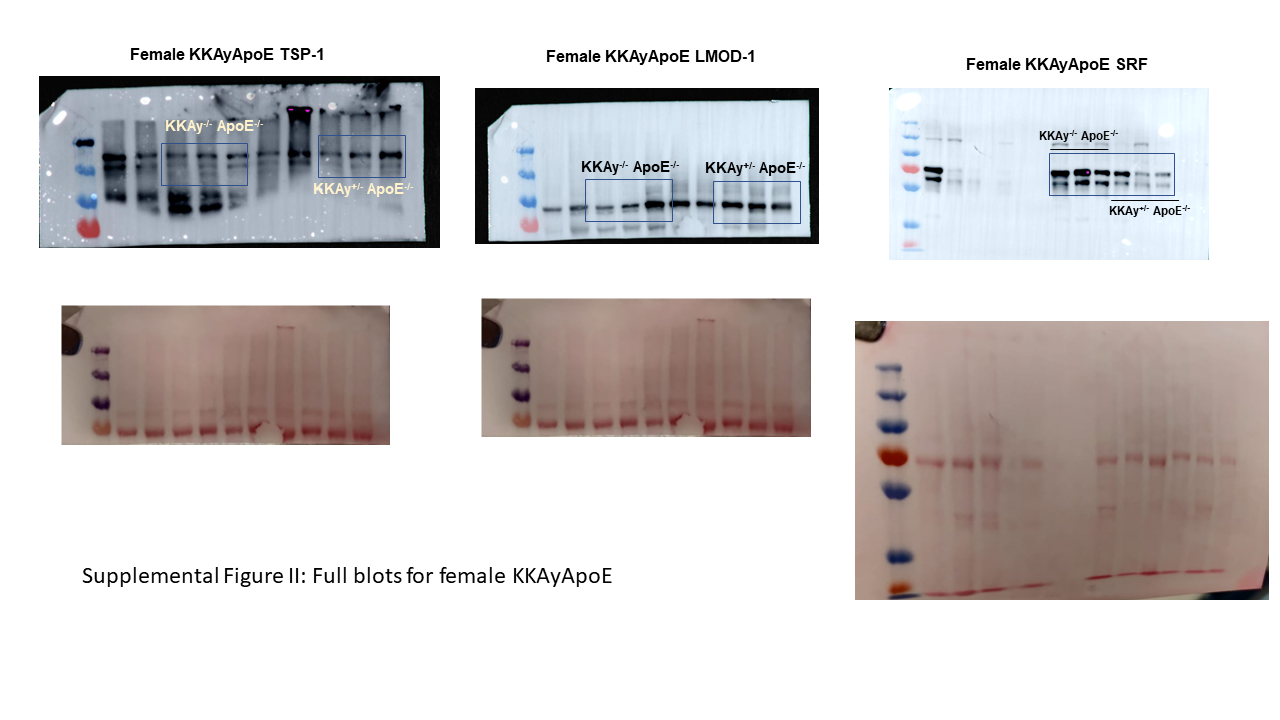

Supplement: Supplementary file 2 [file Image_2.tif]

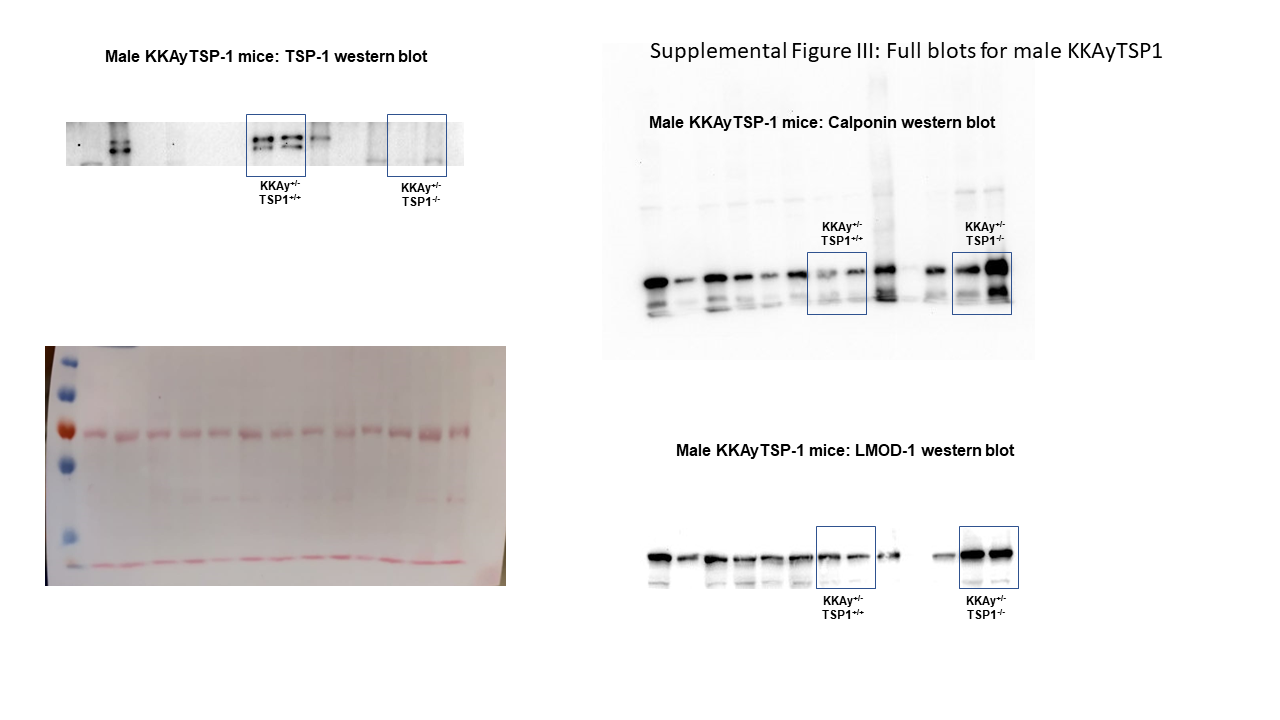

Supplement: Supplementary file 3 [file Image_3.TIF]

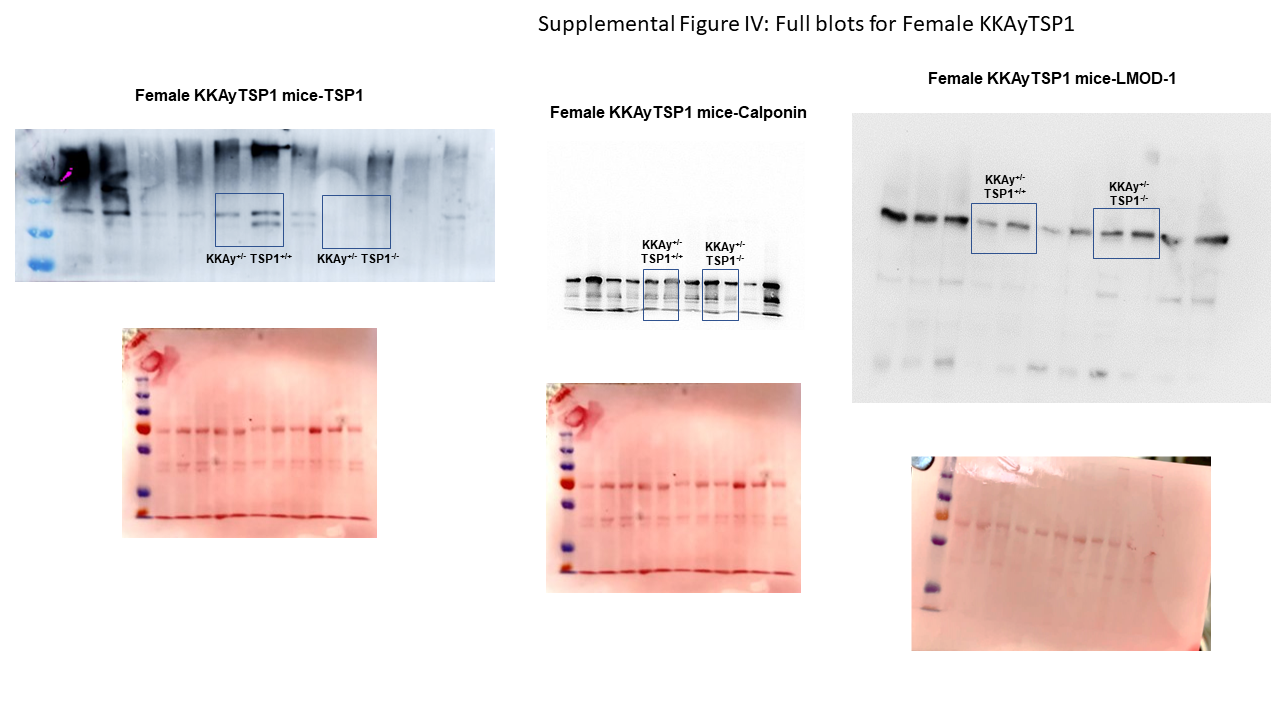

Supplement: Supplementary file 4 [file Image_4.TIF]
